# Reconstruction of Soft Biological Tissues Using Laser Soldering Technology with Temperature Control and Biopolymer Nanocomposites

**DOI:** 10.3390/bioengineering9060238

**Published:** 2022-05-29

**Authors:** Alexander Yu. Gerasimenko, Elena A. Morozova, Dmitry I. Ryabkin, Alexey Fayzullin, Svetlana V. Tarasenko, Victoria V. Molodykh, Evgeny S. Pyankov, Mikhail S. Savelyev, Elena A. Sorokina, Alexander Y. Rogalsky, Anatoly Shekhter, Dmitry V. Telyshev

**Affiliations:** 1 Institute of Biomedical Systems, National Research University of Electronic Technology, Shokin Square 1, Zelenograd, 124498 Moscow, Russia; ryabkin@bms.zone (D.I.R.); molodykh1999@gmail.com (V.V.M.); zugusik@gmail.com (E.S.P.); savelyev@bms.zone (M.S.S.); telyshev_d_v@staff.sechenov.ru (D.V.T.); 2 Institute for Bionic Technologies and Engineering, I.M. Sechenov First Moscow State Medical University, Bolshaya Pirogovskaya Street 2-4, 119435 Moscow, Russia; 3 Department of Oral Surgery of the Institute of Dentistry, I.M. Sechenov First Moscow State Medical Univesity, Bolshaya Pirogovskaya Street 2-4, 119435 Moscow, Russia; lemua@yandex.ru (E.A.M.); prof_tarasenko@rambler.ru (S.V.T.); zabrodalena53@gmail.com (E.A.S.); 4 Department of Experimental Morphology and Biobanking, Institute for Regenerative Medicine, I.M. Sechnov First Moscow State Medical University, Trubetskaya Street 8-2, 119991 Moscow, Russia; fayzullin_a_l@staff.sechenov.ru (A.F.); a.shehter@yandex.ru (A.S.); 5 World-Class Research Center “Digital Biodesign and Personalized Healthcare”, I.M. Sechenov First Moscow State Medical University, Trubetskaya Street 8-2, 119991 Moscow, Russia; 6 OKB BULAT Ltd., Panfilovsky Prospekt 10, Zelenograd, 124489 Moscow, Russia; alexander@laser-bulat.ru

**Keywords:** biological tissues, surgery, recovery, laser weld, temperature feedback, biopolymer structures, carbon nanotubes, regeneration, soldering, epithelization, histology, immunohistochemistry, tensile strength, thin cosmetic scar

## Abstract

Laser soldering is a current biophotonic technique for the surgical recovery of the integrity of soft tissues. This technology involves the use of a device providing laser exposure to the cut edges of the wound with a solder applied. The proposed solder consisted of an aqueous dispersion of biopolymer albumin (25 wt.%), single-walled carbon nanotubes (0.1 wt.%) and exogenous indocyanine green chromophore (0.1 wt.%). Under laser exposure, the dispersion transforms into a nanocomposite due to the absorption of radiation and its conversion into heat. The nanocomposite is a frame structure of carbon nanotubes in a biopolymer matrix, which provides adhesion of the wound edges and the formation of a strong laser weld. A new laser device based on a diode laser (808 nm) has been developed to implement the method. The device has a temperature feedback system based on a bolometric infrared matrix sensor. The system determines the hottest area of the laser weld and adjusts the current supplied to the diode laser to maintain the preset laser heating temperature. The laser soldering technology made it possible to heal linear defects (cuts) in the skin of laboratory animals (rabbits) without the formation of a fibrotic scar compared to the control (suture material). The combined use of a biopolymer nanocomposite solder and a laser device made it possible to achieve a tensile strength of the laser welds of 4 ± 0.4 MPa. The results of the experiment demonstrated that the addition of single-walled carbon nanotubes to the solder composition leads to an increase in the ultimate tensile strength of the laser welds by 80%. The analysis of regenerative and morphological features in the early stages (1–3 days) after surgery revealed small wound gaps, a decrease in inflammation, the absence of microcirculatory disorders and an earlier epithelization of laser welds compared to the control. On the 10th day after the surgical operation, the laser weld was characterized by a thin cosmetic scar and a continuous epidermis covering the defect. An immunohistochemical analysis proved the absence of myofibroblasts in the area of the laser welds.

## 1. Introduction

Laser technologies are widely used in modern surgery. Laser radiation has several advantages over traditional methods (suture material, staples, fibrin glue) due to a specific effect on biological tissues. Laser systems, with the high energy of the generated radiation, are necessary for surgical removal, mechanical destruction or thermal necrosis of cells, tissues or other objects to be eliminated [1]. The possibility of a high concentration of light energy in small volumes allows to selectively affect the biological tissue and regulate the degree of this impact from tissue coagulation to evaporation [2]. This non-contact removal of selected tissues is carried out with high precision and with minimal trauma to the organs. Laser radiation removes the selected areas of the tissue under constant visual control, without damaging the surrounding healthy tissues.

In addition to the mentioned positive effects of laser radiation on biological tissues, it is possible to repair damaged biological tissues using laser welding and soldering technologies [3,4,5,6,7]. Such techniques help to waterproof the welded area (tightness of the wound), seal small vessels and thereby reduce the duration of bleeding and avoid tissue compression and marginal necrosis, so the tissue heals without creating a rough scar. Laser methods are non-contact, so the risk of infection entering the wound is minimal. Compared to other non-contact methods (ultrasound and electric welding), the use of fiber optics in the case of laser methods allows endoscopic and laparoscopic surgery.

The key disadvantages of laser treatment are excessive thermal damage to tissues [8] and the poor tensile strength of the welds, inferring to traditional suture techniques using suture material [9].

The degree of tissue thermal necrosis depends on the characteristics of the laser, such as the radiation power, the geometrical dimensions of the beam exposing to the biological tissues, the soldering method, the time of exposure and the resulting tissue heating temperature [10,11,12,13,14]. In order to minimize thermal damage, each type of biological tissue needs to be adapted to the individual characteristics. Histological studies of the laser-exposed tissue have demonstrated that, if the laser irradiation is correctly selected, there is vaporization of the surface layer of the tissue cells and no thermal damage can be detected [15,16]. For example, there are limitations associated with a high fluid level during laser mucosal reconstruction techniques. In this case, laser exposure leads to inflammation and shrinkage of the mucous membranes, which do not fully recover [17]. The higher the tissue absorption coefficient, the greater the photothermal effect of the irradiation. In this case, the penetration depth of the laser irradiation will be limited and soldering deeper layers of tissue will be difficult. With low tissue absorption coefficients, the laser irradiation may penetrate deeper, but the photothermal effect will be weaker and the tensile strength will be lower.

One of the first lasers used in surgery is the neodymium-doped yttrium aluminum garnet (Nd:YAG) laser, with a wavelength of *λ* = 1064 nm. This wavelength coincides with the absorption peak of melanin and hemoglobin, and thus has a hemostatic effect on soft tissue [18]. The Nd:YAG laser radiation is poorly absorbed by water, so it is allowed to penetrate the tissue at depths of more than 5 mm. The main advantage of using a Nd:YAG laser is the ability to penetrate deep into the biological tissue, but excessive energy density can cause deep uncontrolled thermal damage to the surrounding tissue. Therefore, the use of the Nd:YAG laser in microsurgery is limited [19].

In contrast, the carbon dioxide (CO_2_) laser with a wavelength of *λ* = 10,600 nm is only used in microsurgery. The wavelength of the CO_2_ laser corresponds to the absorption peak of the water. Because water is the main component of most tissues, most of the laser energy is absorbed in the outer layers of the tissue [9,20].

Diode lasers with different wavelengths are the most widely used lasers for welding and soldering biological tissues. Semiconductor lasers with a wavelength of *λ* = 810 nm have a range of advantages due to their small size, low power consumption and the low cost of components compared to other types of lasers. Diode laser radiation can be effectively injected into a fiber optic line, critically important for endoscopic surgery applications [19]. However, the use of diode lasers may have limitations because the peak output power of the radiation is lower than that of CO_2_ and Nd:YAG lasers. This disadvantage is minimized by using special substances applied to the wound area—solders. One significant function of a solder is to increase tissue absorption in the wound area [21,22,23,24]. Laser soldering uses bioorganic solders that are applied to the edges of wounds of various organs [25,26]. The solder intensively absorbs the laser radiation, thus contributing not only to the initial bonding effect of the wound edges but also localizing the laser radiation, reducing thermal tissue damage around the formed laser welds.

Most bioorganic solders used are based on an aqueous suspension of albumin protein [27,28]. The addition of albumin improves the adhesion between tissues, increasing the tensile strength of welds [29]. The thermal stability of albumin allows prolonged heating during laser irradiation. Due to its binding properties, albumin can reduce inflammation and thrombus formation in the soldering area (especially in vessel soldering) [30,31].

Optical absorbers are used to enhance the degree of absorption in the region of the corresponding laser wavelength [32]. This allows laser energy to be concentrated and converted in the area of the weld, avoiding overheating of peripheral healthy tissue. Solutions of indocyanine green (ICG), with a peak absorption in the region of 800 nm, are used for common wavelengths of laser radiation in the near-infrared range in surgery [33].

A tensile strength of 0.027 ± 0.03 MPa is achieved in laser soldering of biological tissue by diode laser (*λ* = 970 nm) without using a solder [34]. However, laser soldering of the same biological tissue by a diode laser (~*λ* = 810 nm) combined with a membrane on a polyester non-woven substrate impregnated to organic solders with different concentrations of bovine serum albumin (BSA) (2 wt.% BSA and 0.02 mg/mL ICG and 40 wt.% BSA and 0.02 mg/mL ICG) achieved a tensile strength of 0.08 ± 0.02 MPa [34] and 0.138 ± 52 MPa [23], respectively. Thus, the addition of a solder during the laser reconstruction increases the strength of the laser weld.

Although the use of protein solders greatly increases the efficiency of laser soldering, the strength of the repaired tissue is not as strong as that of traditional sutures formed with suture material [35]. The use of carbon nanotubes (CNT) in a biopolymer solder is an effective solution to this challenge. CNT have found wide application in medical diagnostics and regenerative medicine due to their size, which corresponds to the size of the main components of the cell matrix, and CNT have properties comparable to protein structures. Nanocomposite structures based on biopolymers and CNT have low cytotoxicity and have a positive effect on cell differentiation and proliferation [36,37]. Although nanotubes tend to aggregate into bundles under van der Waals forces, an aqueous suspension of albumin is an effective dispersant [38]. Under laser irradiation, the dispersion forms a stable nanocomposite material with a framework structure of nanotubes, which improves the suture strength and the efficiency of self-organization of the cells of the repaired tissue [39].

Another way to reduce the area of thermal necrosis of the surrounding biological tissues is to use temperature feedback for the continuous temperature monitoring during tissue repair. To achieve the maximum weld strength and minimum thermal damage, the soldering working temperature should be kept between 50 and 55 °C [40]. In the presented study, the laser system includes temperature feedback, varying the laser power to maintain a constant temperature in the weld formation area.

This paper proposes a technology for the laser reconstruction of soft tissues using a laser system with a wavelength of radiation *λ* = 808 nm, equipped with feedback for the continuous control of temperature, and a nanocomposite solder. The laser exposure of the solder produces a nanocomposite material with a branched carbon nanoframework, providing the formation of a strong laser weld. This method was verified by in vivo experiments with tensile strength measurements and microscopic and histological studies of the welds. This work proves, for the first time, the advantages of the laser soldering technology using a solder based on BSA, ICG and single-walled carbon nanotubes (SWCNT) in combination with the laser device equipped with a new temperature feedback system in in vivo experiments. The temperature feedback system contains an infrared matrix sensor that reads the temperature from a specified area of the laser weld, identifying the pixel corresponding to the most heated point of the laser weld. This system and the developed software are used to maintain the temperature with an accuracy of 0.5 °C of the laser suture, excluding thermal necrosis of tissues. The healing of linear skin defects without fibrous scar formation as compared to traditional sutures, reduction in inflammation, absence of microcirculatory disturbances of blood circulation, earlier epithelialization of laser welds as compared to traditional sutures and increased tensile strength of laser welds were demonstrated. The epithelium in the area of the laser welds with BSA+ICG+SWCNT was almost indistinguishable from the native epidermis 10 days after surgery. The results of the work will allow to bring the technology of laser soldering closer to clinical practice.

## 2. Materials and Methods

This section includes a description of the laser soft-tissue reconstruction system, the component composition of the solder with a detailed description of all substances used and the technology for preparing the solder. The section also describes the algorithm for in vivo laser soldering of soft tissues in laboratory animals. It also presents methods for investigating the structural, mechanical and biomedical characteristics of laser welds in comparison with conventional sutures.

### 2.1. Laser System

For laser soldering operations, a special system was developed, which consisted of following basic modules. The optical module generates and delivers the laser radiation, the temperature module measures and maintains the temperature (temperature feedback) of the weld and the control module provides the necessary parameters for the laser soldering procedure.

The optical module is based on a semiconductor gallium aluminium arsenide (GaAlAs) laser generating continuous wavelength radiation *λ* = 808 ± 3 nm. Wavelengths in the near-infrared spectrum are widely used for laser surgical procedures [21]. The maximum allowable power of laser radiation is 5 W. The laser light is transported to the weld formation area by 600 µm optical fiber. A plane-parallel beam of radiation is formed by a collimator with focal length f = 10.99 mm. The diameter of the laser beam is ~2 mm.

The temperature module is necessary to minimize the risk of thermal necrosis of the soft tissues. For this purpose, the set temperature in the weld formation area is continuously monitored and maintained (temperature feedback).

For measuring soft-tissue temperature during laser reconstruction, the following limiting factors must be considered: sharp temperature contrast between the laser-exposed areas and the surrounding healthy tissue (i), small measuring area (about 2 mm in diameter) (ii) and sharp heating and cooling of the measured surface (iii). In order to achieve maximum temperature measurement accuracy in the area of weld formation and to reduce the influence of limiting factors, the developed laser system uses a bolometric infrared (IR) matrix temperature sensor with 4 × 16 pixels resolution. The maximum value from the temperature matrix is selected, and the laser power is regulated. This algorithm eliminates the error caused by the distance between the optical axes of the laser and the bolometric IR matrix sensor from the weld. This method makes it possible to achieve an error in temperature control—0.5 °C.

To maintain the set temperature, the laser power is regulated by a proportional–integral–differential (PID) regulator. The PID regulator gradually reduces the power supplied to the heater as the set-point temperature is reached. In our case, this is the current supplied to the laser diode by the power source. After the temperature has stabilized, the emitted power *N*, expressed as a percentage of its maximum output, calculated by the Equation (1) [41]:

(1) $$N\left(t\right)=P+I+D=K_{p}e\left(t\right)+K_{i}\int_{0}^{t}e\left(\tau \right)d\tau +K_{d}\frac{de}{dt},$$

where *K*_p_, *K*_i_, *K_d_* are the amplification coefficients of the proportional, integral and differential power regulation components, *P* is the proportional component directly proportional to the difference between the set temperature value and the actual measured temperature value, *I* is the integral component equal to the amount of thermal power required to compensate for heat losses at *∆T* = 0 and *D* is the differential component proportional to the rate of temperature change with the opposite sign and preventing extreme changes in object temperature. In laser soft-tissue reconstruction, all coefficients must be selected individually.

**Figure 1:**
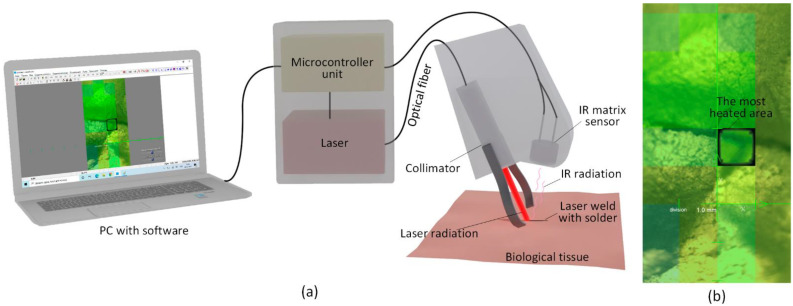
Schematic representation of a soft-tissue laser soldering system (**a**), display with temperature distribution over the area exposed to laser radiation in the software (**b**).

Figure 1a shows a schematic representation of the laser system. In this way, the heating temperature of the laser weld was maintained to the accuracy of 0.5 °C. The laser beam coming out of the fiber with a collimator (1) was directed onto the biological tissue (2). The temperature was recorded using the bolometric IR matrix sensor (3) from the selected area (4). The IR matrix sensor detected the hottest point of the scanned area. The temperature of the hottest point was transmitted to the microcontroller unit, which regulated the current of the laser diode. The required weld heating temperature is set before the operation by a specially developed software for controlling the system. An image of the temperature distribution over the area exposed to the laser light is shown in Figure 1b. The software allows continuous monitoring of the dynamic variation of the weld’s temperature and the laser radiation output. However, it is necessary to perform calibration studies for a particular type of biological tissue and laser solder composition in advance. The selection of PID regulator coefficients providing the fastest temperature setting and its precise maintenance was made with the help of algorithm.

### 2.2. Biopolymer Nanocomposite Solder

In order to carry out laser soldering, a biopolymer nanocomposite solder was developed consisting of BSA (99% purity, BioClot, Aidenbach, Germany) with a concentration of 25 wt.%, ICG (Sigma-Aldrich, St. Louis, MO, USA) with concentration 0.1 wt.%, SWCNT (Institute of Problems of Chemical Physics RAS, Chernogolovka, Russia) with concentration 0.1 wt.% and distilled water. Nanotubes were synthesized by the electric arc method on a nickel-yttrium (Ni/Y) catalyst, purified in air with washing with hydrochloric acid (HCl) and functionalized with carboxyl groups in a nitric acid (HNO_3_) and sulfuric acid (H_2_SO_4_) mixture, with washing until neutral. The average nanotube diameter was 1.4–1.8 nm, the length was ~0.3–0.8 μm and the specific surface area of the product was 400 m^2^/g. A total of 4 types of bioorganic solders were created. The component composition of each group is shown in Table 1. The concentration of solder components was chosen based on previous in vitro studies of the effects of BSA, ICG and SWCNT concentrations on the tensile strength of biological tissues [33,38,42,43].

Several steps were carried out to produce the biopolymer nanocomposite solder. Initially, a SWCNT homogeneous aqueous dispersion was prepared using an ultrasonic homogenizer at 40 W for 45 min. This step was necessary to distribute the nanotubes homogeneously over the volume of water. Nanotubes have hydrophilic properties due to the functionalization with carboxylic groups. Then, ICG and BSA were added to the SWCNT dispersion with continuous mechanical stirring for 30 min. After that, the nanocomposite solder is placed in an ultrasonic bath at 5 W for 45 min to achieve complete homogeneity of the solder. The obtained nanocomposite solder can be stored at 4 °C. 

### 2.3. Laser Soldering Process

Laser soldering has been used to reconstruct the skin integrity of laboratory animals —Chinchilla rabbits. In vivo experiment was carried out under a strict protocol at I.M. Sechenov First Moscow State Medical University under the guidance of its Ethics Committee no. 19-21. Laboratory animals were divided into 5 groups (groups 0–4). Each group had 6 experimental specimens of traditional sutures and laser welds. The wool was shaved on the withers of the animals and markings were made to locate the linear wounds. The skin was dissected with a surgical scalpel. In group 0 animals, the edges of the linear wounds were connected using traditional sutures, i.e., sutured with one of the most common Prolene 5.0 sutures (Ethicon Inc., Bridgewater, NJ, USA). In groups 1–4, the edges of the linear wounds were connected using laser solder according to Table 1. The animals were euthanized by the injection of a solution of ZOLETIL 100 (VIRBAC, Carros, France; 60 mg/kg of animal body weight) at days 1, 3, 7 and 10 after the operation. The excised tissues were studies by histology and immunohistochemistry. The tensile strength of the welds was also investigated. The structure of welds and solders in the solid-phase state was investigated by laser scanning microscopy (LSM) and scanning electron microscopy (SEM).

### 2.4. Scanning Electron Microscopy 

The study of laser solders in solid state (after laser exposure) was carried out by SEM. The FEI Helios NanoLab 650 (FEI Ltd., Hillsboro, OR, USA) was used for this purpose. SEM of the sample layers was carried out at a low vacuum (from 1 to 270 Pa) and low accelerating voltage of electron column (3–25 kV) modes, which are used for biological samples. It was necessary to prevent the samples being destroyed by the primary electron beam and to reduce the effect of charge build-up on the surface. The samples were attached to electrically conductive carbon tape and silicon wafers to allow the charge to flow away.

### 2.5. Laser Scanning Microscopy

Laser welds studies by laser scanning confocal microscopy were performed on an LSM780 microscope (Carl Zeiss, Berlin, Germany). Scans were performed using three excitation wavelengths of 405, 488 and 561 nm argon and diode lasers. Radiation detection was carried out in the wavelength range of 400–750 nm.

### 2.6. Histological and Immunohistochemical Studies 

Four μm thick sections of the formalin-fixed-paraffin-embedded tissue samples were stained with hematoxylin and eosin. A LEICA DM4000 B LED microscope, equipped with a LEICA DFC7000 T digital camera running under the LAS V4.8 software (Leica Microsystems, Wetzlar, Germany) was used for the examination and imaging of the samples. The histological findings indicating inflammatory (wound gap, immune cell infiltration, necrosis) and regenerative (granulation tissue volume, granulation tissue maturity, epithelialization) transformation of the wound were evaluated using 0 to 4 semiquantitative scoring (0—normal tissue, 1—minimal changes, 2—moderate changes, 3—significant changes, 4—maximal registered changes). Experimental data analysis was carried out with the standard software package GraphPad Prism version 8.00 for Windows (GraphPad Software, Inc.). Intergroup differences in the intensity of inflammation and regeneration were analyzed using the Kruskal–Wallis test with the post hoc Dunn’s test. The results for the intensity of inflammation and regeneration were presented as column graphs with mean values (95% confidence intervals). The significant level of differences p was assessed at the value < 0.05.

For immunohistochemical analysis, four μm thick sections of the formalin-fixed-paraffin-embedded tissue samples were deparaffinized, incubated with 3% hydrogen peroxide (H_2_O_2_) for 10 min, underwent heat-induced epitope retrieval (pH 6.0 sodium citrate buffer, 30 min in 80 °C water bath), additionally blocked with Background Block (Cell Marque, Rocklin, CA, USA) and incubated with mouse monoclonal primary antibodies against α-smooth muscle actin (α-SMA) (A2547, Merck Millipore, Burlington, MA, USA, diluted 1:400) and detected by HRP-conjugated secondary goat antibodies (G-21040, Invitrogen, Carlsbad, CA, USA, diluted 1:1000) and diaminobenzidine (DAB) with hematoxylin counterstaining.

### 2.7. Tensile Strength of Welds

The strength sutures and welds investigation were carried out with the 858 Mini Bionix II tensile testing machine (MTS Systems, Eden Prairie, MN, USA). The test specimens were skin sections with rectangular welds, with an aspect ratio of 1:4. Both laser welds and conventional sutures were in the middle of the specimens, perpendicular to the largest of the sides. Two triangular cuts were made in the specimens coaxial to the sutures so that the width of the specimen between the two cuts was equal to the width of the weld. The specimens were attached to the tensile machine by a larger edge area. The welds were placed perpendicular to the tensile force.

The tensile force increased linearly during the test. The tensile strength was calculated at the maximum value of the tensile force. The geometric dimensions of the weld cross-section were measured with an electronic caliper to an accuracy of 10^−5^ m. The tensile strength was set to an accuracy of 10^−2^ N. The tensile strength of the welds was calculated as the ratio of the maximum tensile strength to the cross-sectional area.

## 3. Results and Discussion

This section presents the results of a comparison of the spectral characteristics of solders with different compositions and providing different structures of solid nanocomposites during laser formation. The peculiarities of the micro- and nanostructures of the laser welds using SEM and LSM are demonstrated. The results of the histological and immunohistochemical analyses and the tensile strength of laser sutures are also presented.

### 3.1. In Vivo Studies

**Figure 2:**
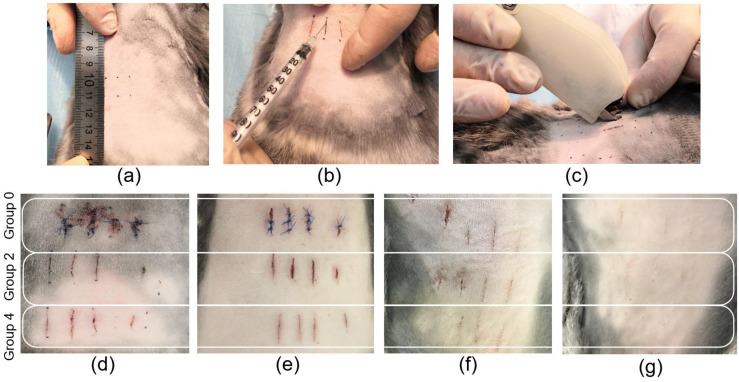
Photos of rabbit skin reconstruction in vivo: marking for dissection (**a**), solder application to the wound (**b**), laser formation of the weld (**c**). Photos of traditional suture (group 0) and laser welds BSA+ICG solder (group 2) and BSA+ICG+SWCNT (group 4) on 1 (**d**), 3 (**e**), 7 (**f**), 10 (**g**) postoperative days.

For the experiments, the location of the linear wounds was marked on laboratory animals and the skin tissue was dissected using a scalpel (Figure 2a). After that, the edges of the linear wounds were connected using suture material to form traditional sutures. For laser welds formation, the solder was applied to the wound area and laser system operated (Figure 2b,c). The procedure (laser soldering) lasted no longer than 1 min for each weld up to 1 cm in length. All the procedures for the integrity recovery of the linear wounds of the tissue were performed under identical conditions. The sutures were divided into five groups (Table 1). However, Figure 2d–g show the appearance of three groups of sutures: traditional sutures (group 0) and laser welds with BSA+ICG (group 2) and BSA+ICG+SWCNT (group 4), because the appearance of the laser welds from groups 1 and 2 and groups 3 and 4 were similar. Figure 2d–g show the recovery process of the linear wounds at different follow-up periods of 1, 3, 7 and 10 days after surgery. The area of the traditional sutures showed the highest amount of blood compared to the laser welds. The visual analysis of the surface structure of the tissue reconstructed by laser soldering showed a smaller scar 10 days after surgery. In a comparison of the BSA+ICG and BSA+ICG+SWCNT solders, the sutures formed with nanotubes appear smaller compared to those without nanotubes.

### 3.2. Laser Solders Structure

**Figure 3:**
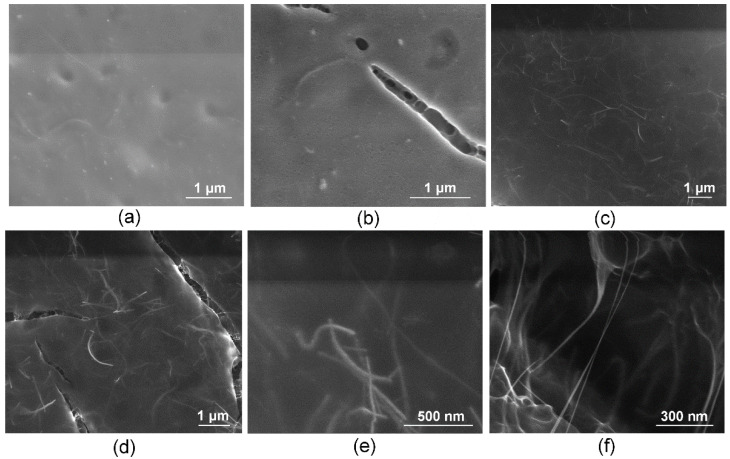
Scanning electron microscopy images of solder based on BSA (group 1) (**a**), BSA+ICG (group 2) (**b**), BSA+SWCNT (group 3) (**c**), BSA+ICG+SWCNT (group 4) (**d**–**f**).

**Figure 4:**
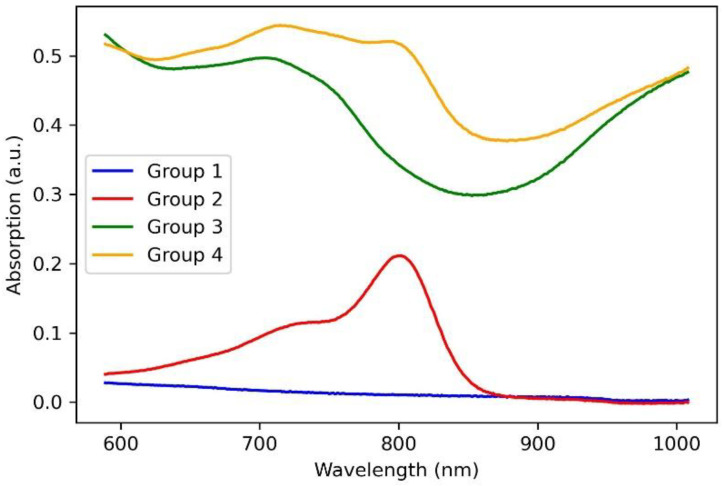
Absorption spectra of solders based on BSA (group 1), BSA+ICG (group 2), BSA+SWCNT (group 3), BSA+ICG+SWCNT (group 4).

The structure of the laser solders from all groups of welds in the solid-phase state (biopolymer nanocomposites) was further studied. The nanocomposites were obtained after laser exposure to the aqueous media in the liquid state and had differences in the internal structure due to different compositions. The nanocomposites were formed under identical laser exposure parameters. To analyze the quality and durability of the laser welds formed using these solders, their microstructure must be investigated, and for solders containing SWCNT, the structure at the nanoscale must be analyzed. It has previously been shown that laser irradiation of nanotubes leads to the binding of carbon nanotubes [44]. Moreover, a laser-induced SWCNT framework structure formation in an albumin- and chitosan-based biopolymer matrix is possible [45]. The laser-induced SWCNT framework provides an increased strength of the biopolymer material [46,47]. The strength is highly dependent on the formed composite structure morphology of the biopolymers and nanotube network [48]. In this regard, the morphology of solders both with and without nanotubes was analyzed by SEM (Figure 3). The surface of the BSA-based solder (group 1) appears homogenous with small indentations (Figure 3a) compared to the BSA-based solder with ICG (group 2). The SEM images of the solder based on BSA (Figure 3a) and BSA+ICG (Figure 3b) are characterized by a lighter image compared to the solder containing the BSA+SWCNT nanotubes (Figure 3c) and the BSA+ICG+SWCNT (Figure 3d–f). The reason for this is that samples without nanotubes exhibit dielectric properties and promote the accumulation of an electronic charge, while samples with the SWCNT have high electrical conductivity, which contributes to the effective flow of charge from the sample to the substrate. Figure 3a,b show elongated fibrillar structures. The structures are parts of the BSA matrix. The surface of this solder has cracks up to a few µm long and up to 250 nm wide (Figure 3b). The appearance of these cracks can be attributed to the greatest local heating due to the increased absorption coefficient of the ICG solder, the wavelength of which corresponds to the maximum absorption spectrum of the ICG solder (Figure 4).

A similar pattern is observed with the other solder pair with the SWCNT—groups 3 and 4 (Figure 3c,d). The BSA+SWCNT nanocomposite shows cracks up to 30 µm long and 3 µm wide, compared to the nanocomposite with the SWCNT but without the ICG [37,38]. The presence of cracks can lead to both positive and negative sides. On the one hand, cracks can reduce the strength of laser sutures. On the other hand, the presence of large cracks affects the acceleration of hard solder bioresorption and wound healing. Because, for effective tissue recovery, porous implants are used to provide the sprouting of cells, nerve fibers and blood vessels. The cracks in the nanocomposites from group 4 are bonded together by a framework of nanotubes, as shown in Figure 3e,f. The nanotubes form contacts with each other in the BSA matrix (Figure 3e). The size of the SWCNT was increased by combining them into bundles and enveloping them in a BSA layer [40].

### 3.3. Sutures Structure 

**Figure 5:**
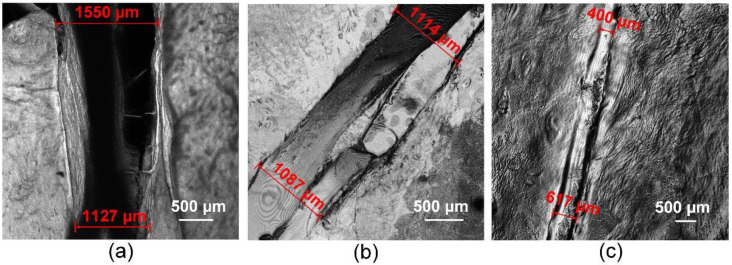
Laser scanning Microscopy images of a traditional suture (**a**) and laser welds with solder based on BSA+ICG (group 2) (**b**), BSA+ICG+SWCNT (group 4) (**c**).

The LSM produced 3D images of the traditional sutures (Figure 5a) and laser welds with the solder from groups 2 and 4 (Figure 5b,c). Welds formed by laser soldering appear more airtight as the area between the edges of the biological tissue is filled with liquid solder. The laser radiation transforms the solder from liquid to solid and connects the edges of the wound. The traditional suture after operation is characterized by a large wound edge distance of up to 1.5 mm. The use of the BSA+ICG-based solders achieved a laser weld width of up to 1 mm (Figure 5b). The use of the BSA+ICG+SWCNT-based solder provides the greatest wound edge closure with a width of less than 1 mm (Figure 5c). The addition of the CNT in the solder composition provides a smaller weld width in comparison to other welds, providing uniformity along the entire length of the weld. The uniformity of the welds strongly affects the tightness of the wound, and the tightness affects the rate of repair of the defect, mostly important in the reconstruction of blood vessels and tissues saturated with blood vessels. Thus, the investigation shows that the addition of the SWCNT as an additional solder component reduced the width of the laser welds formed. Reducing the width of the suture greatly increases the rate of regeneration and recovery of the reconstructed biological tissue functionality.

### 3.4. Histological and Immunohistochemical Analyses

**Figure 6:**
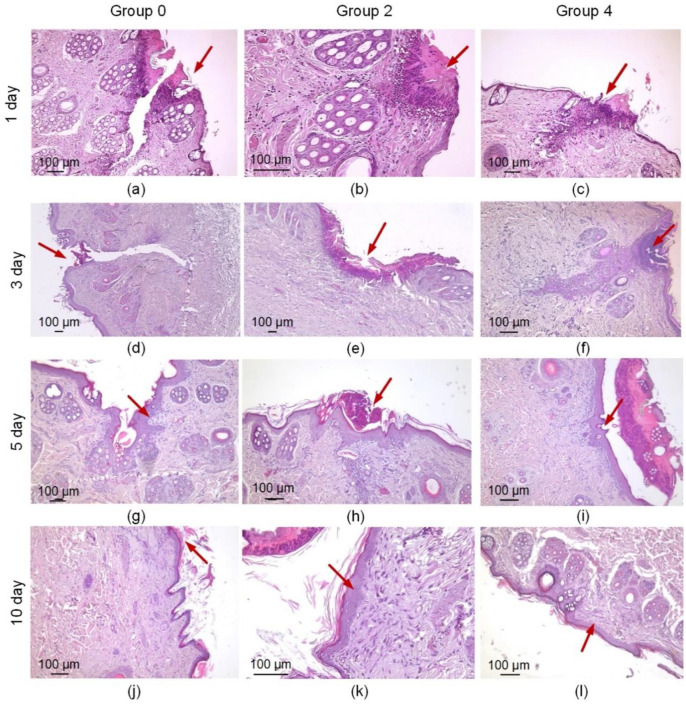
Histological images of the three suture groups: traditional suture (**a**,**d**,**g**,**j**) and laser welds with BSA+ICG solder (group 2) (**b**,**e**,**h**,**k**) and BSA+ICG+SWCNT (group 4) (**c**,**f**,**i**,**l**) on 1, 3, 7, 10 postoperative days. The red arrow points to a formed traditional suture or laser weld.

The histological examination revealed a relatively wide wound gap in group 0 a day after the operation (Figure 6a). The wound contained a sutured thread, around which a moderate inflammatory infiltration was visible, consisting mainly of neutrophils, eosinophils and macrophages (Figure 6a). There was a relatively large area of necrosis of the epidermis and papillary dermis. In group 2, the wound edges were merged using laser radiation and a solder of BSA and indocyanine, and a minor area of coagulation necrosis was observed (Figure 6b). Around the necrosis, there was a wide leukocyte infiltration, consisting of neutrophils and eosinophils with a small admixture of macrophages. In group 4, where the defects were treated using laser radiation and the solder with the BSA+ICG+CNT, there was a relatively small wound gap surrounded by tissues infiltrated by neutrophils and eosinophils. The wound gap was not elongated, and the area of necrosis in group 4 was smaller than in groups 0 and 2 (Figure 6c). In group 4, full-blooded vessels with sludged erythrocytes were found, indicating intensification of the wound healing.

**Figure 7:**
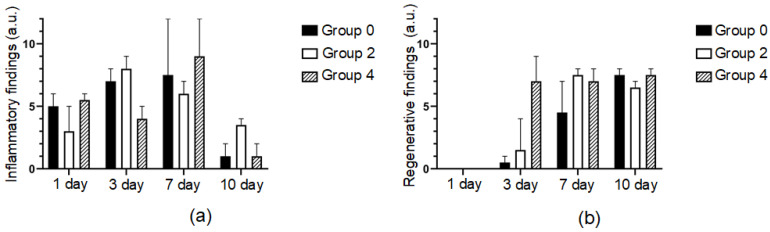
Histograms of inflammatory (**a**) and regenerative (**b**) processes in defect areas treated with traditional sutures (group 0), laser soldering with BSA+ICG (group 2) or BSA+ICG+SWCNT (group 4) on 1, 3, 7, 10 postoperative days. Mean values ± SD.

Three days after the operation, a gap remained at the incision site in all samples in group 0. The fissure passed through the entire thickness of the dermis; the wound was not epithelialized (Figure 6d). On the surface, there were fragments of necrotic epithelium and between two sides of the dermis—a scab. However, proliferating fibroblasts, numerous capillaries and elongated longitudinally arranged collagen fibers with moderate lympho-macrophage infiltration surrounded the gap and filled the bottom with the defect (Figure 7a). This indicated the beginning of the formation of scar tissue. In group 2, the wound gap was either absent or filled with compacted fibrin. The scab was observed on the surface of the wound and consisted of necrotic cells of the epidermis and dermis fibers. The thickness of the scab was higher than in the comparison group 2 (Figure 6e). The immune cell infiltration around the fissure was moderate. Immature granulation tissue formed focally. In one sample, a continuous epithelial layer formed under the scab, consisting of young epidermal cells without a clear division into layers, but with a basement membrane indicating complete epithelialization of the defect (Figure 7b). In group 4, there was no wound gap, but, because the edges of the incision were closely soldered, there was a moderate proliferation of fibroblasts and lympho-macrophage infiltration (Figure 7a). A scab of dried exudate remains above the epithelium (Figure 6f). The surface of the wound was epithelialized in two samples (Figure 7b).

On the 7th postoperative day, the surface of the wound defect was epithelialized only in two samples in group 0. There was no wound gap, and an immature scar was observed. Epithelization was not complete in three other samples: there was space between the two layers of the new epithelium (Figure 7b). A scab and immature scar tissue were closely adjacent to the non-epithelialized surface infiltrated by lymphocytes, macrophages and sparse neutrophils (Figure 6g). In group 2, the defect was epithelized in four samples. The maturity of the epithelium varied: in some samples, the gap was partially filled with dense fibrin, and in other samples, the gap was filled with fibrosing granulation tissue with an increased number of fibroblasts, while immune cell infiltration was minimal (Figure 6h). In group 4, the defect was epithelialized in all five samples. The wound gap was partially filled with fibrin and granulation tissue (Figure 7b). The maturity of the scar tissue was higher than in group 2 (Figure 6i). 

On the 10th day, the defect was completely epithelialized in all samples in group 0. The epithelium was mature and had the same structure as the intact epithelium. At the site of the injury, there was a scar that was relatively wide at the base (under the epithelium) and narrow or absent in the deeper parts of the dermis (Figure 6j). There remained a mild lymphocyte and macrophage infiltration of the defect tissue (Figure 7a). In group 2, the surface of the defects was lined with mature epithelium covered with a scab (Figure 6k). The scar passing through the dermis was very thin and mostly impossible to detect. The scar tissue volume expanded in the subepithelial layer, where it consisted of densely packed collagen fibers and fibroblasts. In group 4, the surface of the defect was epithelized in all samples (Figure 7b). The epithelium practically did not differ from the native epidermis (Figure 6l). In two samples, the scar was very thin, almost imperceptible, and differed only in an increased number of fibroblasts compared to the dermis and weak lympho-macrophage infiltration.

**Figure 8:**
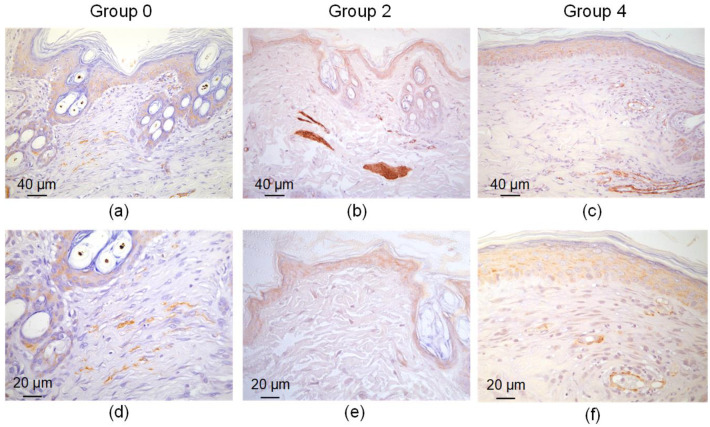
α-SMA expression in defect sites treated with simple sutures (**a**,**d**), laser soldering with BSA+ICG (group 2) (**b**,**e**) or BSA+ICG+SWCNT (group 4) (**c**,**f**) 10 days after the operation.

The scar tissue in group 0 consisted of a densely packed fibrotic fibroblast—myofibroblasts (Figure 8). These cells expressed α-SMA and overproduced collagen. The presence of these cells indicates a continuous fibrotic transformation of the tissue. The wound sites in groups 2 and 4 lacked these cells. α-SMA expression was observed in normal muscular microstructures—artery walls and the muscular apparatus of the hair follicles.

### 3.5. Tensile Strength of Welds

The tensile strength was determined for all groups of sutures and welds formed by the means of the suture material and laser solders. The tensile strength of the native tissue was also measured. The tensile strength of the native tissue was $\bar{\sigma }$_native_ = 8.6 ± 1.2 MPa. Tensile strength studies were performed on days 1, 3, 7 and 10 after surgery. None of the compounds used achieved a tensile strength comparable to the native tissue on day 10. However, the achieved tensile strength is sufficient to maintain the physical integrity of the skin under domestic and everyday loads. With further skin regeneration after day 10, the tensile strength will increase.

**Figure 9:**
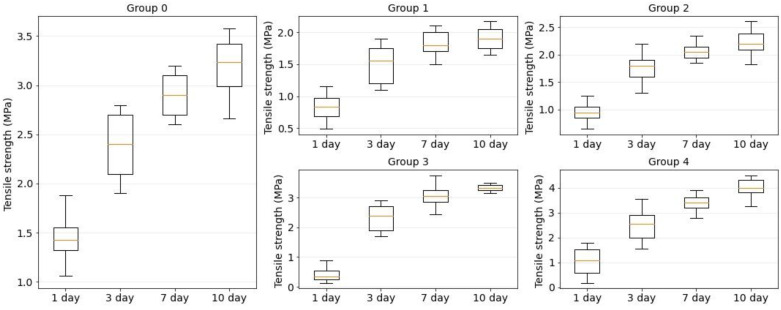
Tensile strength diagrams for traditional suture (group 0) and laser welds with BSA (group 1), BSA+ICG (group 2), BSA+SWCNT (group 3) and BSA+ICG+SWCNT (group 4) on 1, 3, 7, 10 postoperative days.

Figure 9 shows the strength dependence of the laser welds formed using four component solder compositions: BSA, BSA+ICG, BSA+SWCNT and BSA+ICG+SWCNT (groups 1–4). The orange line is the median. The box extends from the first quartile to the third quartile of the tension strength data. The whiskers extend from the box by 1.5 in the interquartile range.

On the first postoperative day, the maximum tensile strength from the laser sutures was detected in the laser weld formed using a solder based on BSA+ICG+SWCNT ($\bar{\sigma }$_BSA+ICG+SWCNT_ = 1 ± 0.6 MPa), but even it was less than the tensile strength of a traditional suture formed using needle thread ($\bar{\sigma }$_suture_ = 1.6 ± 0.3 MPa). On day 10 after surgery, the tensile strength of the laser welds exceeded the traditional welds (group 0). Furthermore, the increase was detected for two solder compositions: BSA+SWCNT ($\bar{\sigma }$_BSA+SWCNT_ = 3.3 ± 0.2 MPa) and BSA+ICG+SWCNT ($\bar{\sigma }$_BSA+ICG+SWCNT_ = 4 ± 0.4 MPa).

According to Figure 9, the recovery of the skin tensile strength was nonlinear. For all investigated welds, the maximum difference in the tensile strength was observed on days 1 and 3 postoperatively, while the minimum difference was obtained on days 7 and 10.

The strength of the laser welds on postoperative days 1 and 3 was significantly lower than the tensile strength of the sutures obtained by the traditional method. However, on the tenth day, laser sutures formed with the BSA+SWCNT (group 2) and BSA+SWCNT+ICG (group 4) showed a higher tensile strength compared to the conventional sutures (group 0).

With carbon nanotubes as an additional solder component, the tensile strength of laser welds exceeded the tensile strength of traditional sutures. Because insufficient tensile strength is one of the main factors limiting the wide application of laser soldering technology in medical practice, the application of the proposed solder in combination with the developed laser system will significantly expand the use of laser methods to reconstruct the integrity of biological tissues.

## 4. Conclusions

Laser soldering technology has been developed for the surgical reconstruction of the integrity of biological tissues. For this purpose, the laser system based on the diode laser with a wavelength of 808 nm has been developed. The system has a temperature feedback module based on a bolometric infrared matrix sensor. The system detects the hottest area of the laser weld and adjusts the current to the laser diode. In this way, the target temperature of the laser weld is maintained with an accuracy of 0.5 °C.

In order to increase the absorption of radiation to prevent the thermal necrosis of tissue and increase the strength of laser welds, this study proposes solders that are applied to the edges of the wound. The solders are an aqueous dispersion of bovine serum albumin, indocyanine green and single-walled carbon nanotubes. During laser irradiation, the liquid dispersion is converted into a nanocomposite. The nanocomposite is a framework structure of carbon nanotubes in a biopolymer matrix, providing wound edge bonding and the formation of a durable laser weld. Electron microscopy showed the formation of the single-walled carbon nanotubes framework simultaneously, increasing their diameter by bundling and enveloping in an albumin layer. It was found that the addition of indocyanine green to the solder composition promotes an increase in absorption and the appearance of cracks up to 30 µm in size in the nanocomposite. This fact contributes to an increase in the rate of the bioresorption of the single-walled carbon nanotubes nanocomposites providing wound recovery. Using laser microscopy, it was found that the addition of the solder reduced the width of the laser sutures formed and increased their uniformity.

In vivo experiments demonstrated healing of linear skin defects without fibrous scar formation compared to traditional sutures (suture material). An analysis of regenerative and morphological features 1–3 days after surgery demonstrated small wound lumens, reduced inflammation, no microcirculatory disturbances and earlier epithelialization of laser welds compared to traditional sutures. On the 10th day after surgery, the laser welds were characterized by a thin cosmetic scar and a continuous epidermis covering the defect. The epithelium in the area of the laser weld with bovine serum albumin, indocyanine green and single-walled carbon nanotubes was almost indistinguishable from the native epidermis. An immunohistochemical analysis confirmed the absence of myofibroblasts in the area of the suture, which overexpress α-SMA and produce collagen.

The combined use of bovine serum albumin, indocyanine green and single-walled carbon nanotubes solder and a laser system resulted in a tensile strength of 4 ± 0.4 MPa for the laser welds at 10 days after surgery, whereas the strength of the native tissue without sutures was 8.6 ± 1.2 MPa. Experimental results showed that the addition of single-walled carbon nanotubes to the solder composition resulted in an 80% increase in the ultimate strength of the laser welds.

In summary, laser soldering with the developed system and solder based on bovine serum albumin, indocyanine green and single-walled carbon nanotubes can be used to reconstruct the integrity of biological tissue more completely and quickly, without causing scarring. Skin recovery in the area of the simulated wound occurs by primary tension with the formation of an imperceptible normotrophic scar according to the results of the in vivo experiments. Thus, it can be assumed that this technology can be useful for reconstructing the integrity of delicate connective tissues, for example, in ophthalmology, gynecology, urology and others.

## Figures and Tables

**Table 1:** Weld parameters.

Group	Reconstruction Technology	Solder Components (wt.%)		
		BSA	ICG	SWCNT
0	Suture material	-	-	-
1	Solder	25	0.0	0.0
2	Solder	25	0.1	0.0
3	Solder	25	0.0	0.1
4	Solder	25	0.1	0.1
